# Muscle-Type Nicotinic Receptor Blockade by Diethylamine, the Hydrophilic Moiety of Lidocaine

**DOI:** 10.3389/fnmol.2016.00012

**Published:** 2016-02-15

**Authors:** Armando Alberola-Die, Gregorio Fernández-Ballester, José M. González-Ros, Isabel Ivorra, Andrés Morales

**Affiliations:** ^1^División de Fisiología, Departamento de Fisiología, Genética y Microbiología, Universidad de AlicanteAlicante, Spain; ^2^Instituto de Biología Molecular y Celular, Universidad Miguel HernándezAlicante, Spain

**Keywords:** diethylamine, lidocaine, nicotinic acetylcholine receptors, *Xenopus* oocytes, microtransplanted receptors, allosteric modulation

## Abstract

Lidocaine bears in its structure both an aromatic ring and a terminal amine, which can be protonated at physiological pH, linked by an amide group. Since lidocaine causes multiple inhibitory actions on nicotinic acetylcholine receptors (nAChRs), this work was aimed to determine the inhibitory effects of diethylamine (DEA), a small molecule resembling the hydrophilic moiety of lidocaine, on *Torpedo marmorata* nAChRs microtransplanted to *Xenopus* oocytes. Similarly to lidocaine, DEA reversibly blocked acetylcholine-elicited currents (*I*_*ACh*_) in a dose-dependent manner (*IC*_**50**_ close to 70 μM), but unlike lidocaine, DEA did not affect *I*_*ACh*_ desensitization. *I*_*ACh*_ inhibition by DEA was more pronounced at negative potentials, suggesting an open-channel blockade of nAChRs, although roughly 30% inhibition persisted at positive potentials, indicating additional binding sites outside the pore. DEA block of nAChRs in the resting state (closed channel) was confirmed by the enhanced *I*_*ACh*_ inhibition when pre-applying DEA before its co-application with ACh, as compared with solely DEA and ACh co-application. Virtual docking assays provide a plausible explanation to the experimental observations in terms of the involvement of different sets of drug binding sites. So, at the nAChR transmembrane (TM) domain, DEA and lidocaine shared binding sites within the channel pore, giving support to their open-channel blockade; besides, lidocaine, but not DEA, interacted with residues at cavities among the M1, M2, M3, and M4 segments of each subunit and also at intersubunit crevices. At the extracellular (EC) domain, DEA and lidocaine binding sites were broadly distributed, which aids to explain the closed channel blockade observed. Interestingly, some DEA clusters were located at the α-γ interphase of the EC domain, in a cavity near the orthosteric binding site pocket; by contrast, lidocaine contacted with all α-subunit loops conforming the ACh binding site, both in α-γ and α-δ and interphases, likely because of its larger size. Together, these results indicate that DEA mimics some, but not all, inhibitory actions of lidocaine on nAChRs and that even this small polar molecule acts by different mechanisms on this receptor. The presented results contribute to a better understanding of the structural determinants of nAChR modulation.

## Introduction

Nicotinic acetylcholine receptors (nAChRs) belong to the “Cys-loop” superfamily of ligand-gated ion channels (LGICs). All members of this family of receptors are constituted by five subunits, each one contributing four transmembrane spanning-segments (M1–M4), which conform a channel pore lined by the M2 segment of each subunit (Albuquerque et al., 2009; Hurst et al., 2013). nAChRs are widely distributed in central and peripheral nervous systems, but they are also expressed in skeletal muscle fibers and other tissues where they play relevant functional roles (Gotti and Clementi, 2004). Although there is a large heterogeneity in the structure and function of nAChRs from different cells, all of them behave as allosteric proteins, undergoing conformational changes (from resting to active or desensitized states) when specific ligands bind to their orthosteric sites (Taly et al., 2009; Cecchini and Changeux, 2015).

nAChRs are involved in the etiopathology of several neurological disorders, including Parkinson's disease, some myasthenic syndromes, addiction, depression, cognitive deficits (including Alzheimer's disease), some types of epilepsy and also seem to contribute to modulate pain or inflammatory responses (Gotti and Clementi, 2004; Dani and Bertrand, 2007; Hurst et al., 2013). Hence, it is important to study the mechanisms of modulation of these receptors by allosteric ligands and to unravel their specific binding sites, in order to develop new therapeutic agents (Arias, 2010; Chatzidaki and Millar, 2015).

Lidocaine is commonly used in clinical practice as a local anesthetic and as an anti-arrhythmic agent because it reversibly blocks voltage-dependent Na^+^ channels (Hille, 1966). Besides, lidocaine modulates the function of other voltage-dependent channels, including Ca^2+^ (Xiong and Strichartz, 1998) and K^+^ channels (Trellakis et al., 2006) and different pentameric LGICs, such as 5-hydroxytryptamine-3 (Ueta et al., 2007), glycine (Gly) and GABA_A_ receptors (Hara and Sata, 2007), nAChRs (Steinbach, 1968; Gentry and Lukas, 2001; Alberola-Die et al., 2011, 2013) and the prokaryotic channels GLIC and ELIC (Hilf et al., 2010; Gonzalez-Gutierrez and Grosman, 2015). However, the specific mechanisms of action of lidocaine on neuroreceptors are not fully understood.

**Figure 1:**
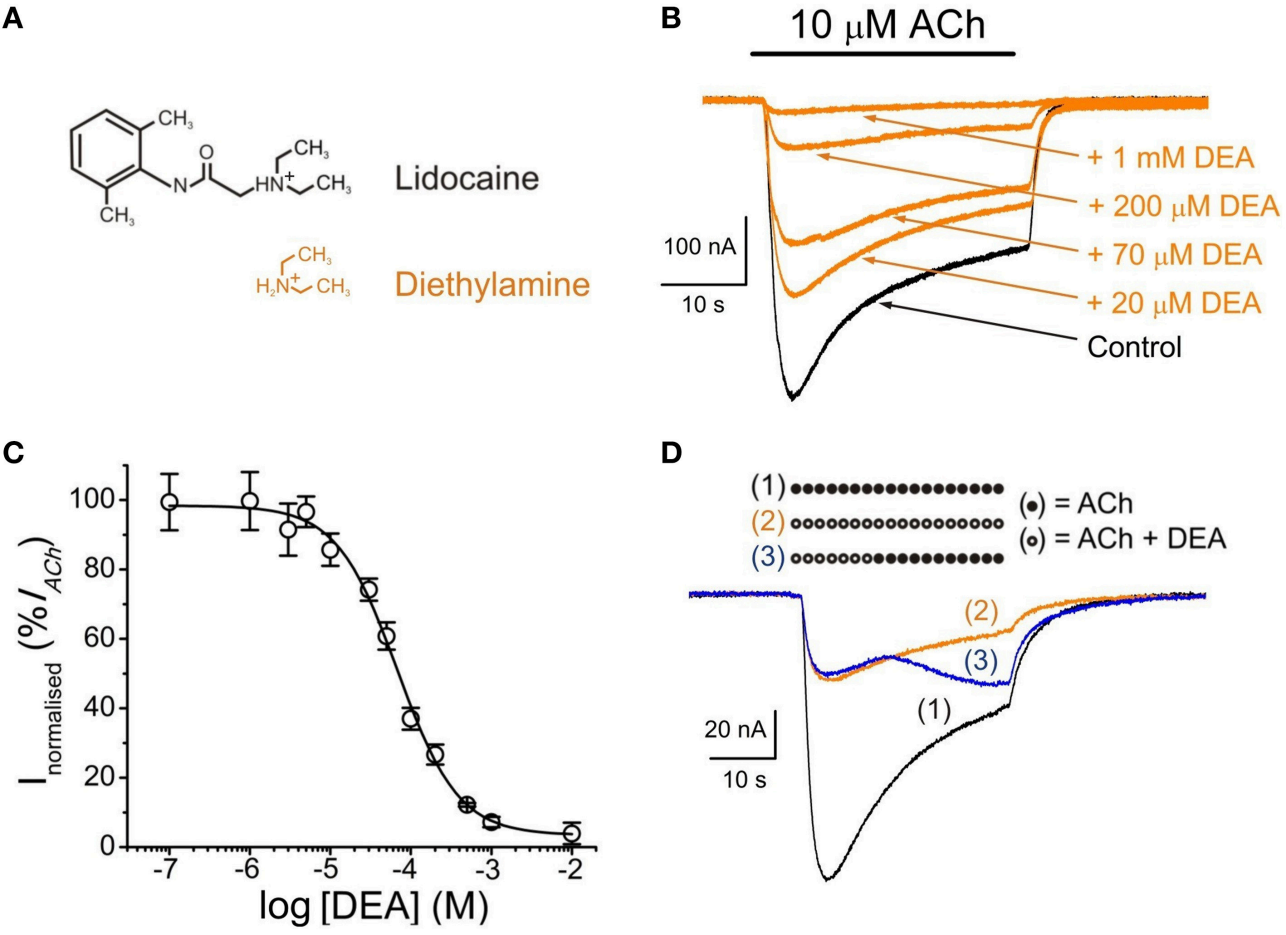
**DEA effects on ACh-induced currents (***I***_***ACh***_s). (A)** Molecular structures of protonated lidocaine and diethylamine (DEA), showing the resemblance of DEA to the hydrophilic moiety of lidocaine. Note that DEA is positively charged at physiological pH, as most lidocaine molecules. **(B)** Superimposed *I*_*ACh*_s recorded in the same nAChR-bearing oocyte by successive applications of 10 μM ACh either alone (Control, black) or together with DEA (orange) at the indicated concentrations. In this and subsequent figures, unless otherwise stated, the holding potential was −60 mV, downward deflections denote inward currents and the horizontal bar above records corresponds to the timing of drug application. **(C)** DEA concentration-*I*_*ACh*_ inhibition relationship. Amplitude of the *I*_*ACh*_s evoked in presence of DEA was normalized to the *I*_*ACh*_ elicited by ACh alone (Control) and plotted vs. the logarithm of DEA concentration. Data are the average of four oocytes from different donors and error bars, in this and following figures, are SEM; solid line is a sigmoid curve fitted to the data. **(D)** Superimposed *I*_*ACh*_s recorded sequentially in the same oocyte by superfusing the cell with 10 μM ACh alone [(1), bar of solid circles and black recording], co-applied with DEA [100 μM; (2), bar of open circles and orange recording] or when changing from ACh plus DEA to ACh alone at the time indicated by the corresponding bar [(3), bar of open circles followed by solid circles and blue recording].

Recently, we have described that lidocaine blocks muscle-type (Alberola-Die et al., 2011) and neuronal heteromeric (Alberola-Die et al., 2013) nAChRs by multiple mechanisms that can be dissected, at least partially, by dose. At doses lower than the *IC*_**50**_, lidocaine inhibits nAChRs mainly by open-channel blockade, whereas at higher concentrations (equal or higher than the *IC*_**50**_) it also causes closed-channel blockade and enhances desensitization. Now, our aim is to unravel the structural determinants within the lidocaine molecule responsible for specific effects on muscle-type nAChRs. So, in this work we have analyzed the effects of diethylamine (DEA), a small molecule resembling the hydrophilic moiety of the lidocaine molecule (see Figure 1A), on nAChRs.

When dealing with ligands bearing different chemical groups in their structure and exhibiting multiple functional effects, it would be ideal to be able to dissect the functional role of individual structural components by using smaller molecules mimicking specific domains of the larger ligand. This is the rationale behind this study on the hydrophilic DEA, which mimics the terminal amine group of lidocaine and indeed reproduces with high fidelity some, but not all, of its effects. Once this has been stablished, it opens the way to use small molecules resembling the hydrophobic moiety of lidocaine to determine which, if any, of the effects of the entire ligand on AChRs function can also be attributed to such hydrophobic domain.

Preliminary results have been published elsewhere (Alberola-Die et al., 2012).

## Materials and methods

### Purification and reconstitution of nAChRs

nAChRs from *Torpedo marmorata* electroplax were purified by bromoacetylcholine-affinity chromatography in the presence of asolectin lipids using cholate as a detergent. After elution with carbamylcholine, purified receptors were dialyzed and reconstituted in asolectin lipids at a final protein concentration of 0.3–1.2 mg ml^−1^. Samples were aliquoted and stored in liquid nitrogen (Ivorra et al., 2002).

### Oocyte preparation and microinjection

Adult female *Xenopus laevis* (purchased from Harlan Interfauna Ibérica S.L., Barcelona, Spain; and Centre National de la Recherche Scientifique, Montpellier, France) were immersed in cold 0.17% MS-222 for 20 min and a piece of ovary was drawn out aseptically. Animal handling was carried out in accordance with the guidelines for the care and use of experimental animals adopted by the E.U. and the animal protocol was approved by the ethic committee of Universidad de Alicante. Stage V and VI oocytes were isolated and their surrounding layers removed manually. Cells were kept at 15–16°C in a modified Barth's solution [88 mM NaCl, 1 mM KCl, 2.40 mM NaHCO_3_, 0.33 mM Ca(NO_3_)_2_, 0.41 mM CaCl_2_, 0.82 mM MgSO_4_, 10 mM HEPES (pH 7.4), 100 U ml^−1^ penicillin, and 0.1 mg ml^−1^ streptomycin] until used. Oocytes were microinjected with 100 nl of an aliquot of reconstituted nAChRs (Morales et al., 1995).

### Two-electrode voltage-clamp recordings in oocytes

Membrane current recordings were performed at 21–25°C, 16–72 h after proteoliposome injection, using a high compliance two-microelectrode voltage-clamp system (TurboTEC-10CD, npi Tamm, Germany). The recording methodology has been described previously (Morales et al., 1995; Olivera-Bravo et al., 2005). Briefly, intracellular microelectrodes (0.8–3 MΩ) were filled either with 3 M KCl or potassium acetate for voltage recording and current injection, respectively. Oocytes were placed in a 150 μl recording chamber and continuously superfused with normal frog Ringer's solution (NR: 115 mM NaCl, 2 mM KCl, 1.8 mM CaCl_2_, 5 mM HEPES, pH 7.0) supplemented with 0.5 μM atropine sulfate (normal Ringer with atropine, ANR) to block any muscarinic response (Kusano et al., 1982). The membrane potential was held at −60 mV, unless otherwise stated. ACh and other tested drugs were diluted in ANR solution and oocytes were superfused with them at a flow rate of 13–17 ml min^−1^. Membrane currents elicited by ACh (*I*_*ACh*_) either alone or co-applied with DEA, were low-pass filtered at 30–1000 Hz and, after sampling at fivefold the filter frequency (Digidata 1200 Series and Digidata 1440A; Axon Instruments, Foster City, CA, USA), recorded on two PC-computers, using the WCP v.3.2.8 package developed by J. Dempster (Strathclyde Electrophysiology Software, University of Strathclyde, Scotland, UK) and AxoScope v. 10.0.0.60 (Molecular Devices Corporation, Sunnyvale, U.S.A.).

### Experimental design

DEA concentration-*I*_*ACh*_ inhibition relationship was determined by measuring *I*_*ACh*_s evoked by 10 μM ACh alone or together with different DEA concentrations. For competition assays, ACh concentration-*I*_*ACh*_ amplitude curves were obtained by exposing injected oocytes to increasing ACh concentrations, either alone or together with 100 μM DEA. *I*_*ACh*_s recorded in the presence or absence of DEA were normalized to the *I*_*ACh*_ evoked by 1 mM ACh alone and fitted to a sigmoid curve. To allow nAChRs to recover from desensitization, the interval between consecutive ACh applications was at least 5 min. To assess the blockade of resting nAChRs by DEA, we compared the *I*_*ACh*_s elicited by ACh (from 1 μM to 1 mM) alone or co-applied with 100 μM DEA either directly or after pre-application of DEA (same concentration) for 12 s.

When studying the voltage dependence of the *I*_*ACh*_ blockade by DEA, series of 800 ms voltage pulses (in 10 mV steps from −120 to −20 mV, followed by 20 mV jumps from −20 to +60 mV; occasionally, in 20 mV steps from −120 to +60 mV) were given to the oocyte before ligand superfusion and during the *I*_*ACh*_ plateau elicited by 10 μM ACh, either alone or co-applied with DEA at different concentrations. In a few cells, the −120 mV pulse duration was extended up to 1500 ms to allow a more complete current relaxation.

### Data analysis and statistical procedures

Inhibition curves were determined by measuring *I*_*ACh*_ evoked by 10 μM ACh in the presence of different DEA concentrations. *I*_*ACh*_s elicited in the presence of DEA were normalized to the *I*_*ACh*_ evoked by ACh alone. Data were fitted to a single-site inhibition curve using the Origin 6.1 software (OriginLab Corp. Northampton, MA, U.S.A.).

Reversibility of *I*_*ACh*_ blockade by DEA was determined by giving 32 s pulses of ACh either alone or co-applied with DEA, for solely the first 12 s or during the whole pulse; *I*_*ACh*_ recovery was measured 20 s and 7 min after DEA washout. The percentage of recovery from blockade (% Recovery) was obtained using the equation:

(1) $$\begin{array}{l} \%\;Recovery=\left[\left(I_{ACh\;after\;DEA}-I_{ACh+DEA}\right)∕\left(I_{ACh}-I_{ACh+DEA}\right)\right]\times 100 \end{array}$$

where *I*_*ACh*_ is the current amplitude evoked by 10 μM ACh alone; *I*_*ACh*+*DEA*_, is the current elicited by co-application of 10 μM ACh with 100 μM DEA; and *I*_*ACh after DEA*_ is the current obtained 20 s or 7 min after DEA removal.

The rate of desensitization was determined by measuring the *I*_*ACh*_ amplitude elicited by 100 μM ACh, either alone or co-applied with up to 200 μM DEA, at different times after *I*_*ACh*_ peak. Desensitization rates were obtained using the equation:

(2) $$\begin{array}{l} D_{ti}=\left[1-\left(I_{ti}∕I_{peak}\right)\right]\times 100 \end{array}$$

where *D*_*ti*_ is the desensitization value at the specified time; *I*_*peak*_ the *I*_*ACh*_ amplitude at the peak; and *I*_*ti*_ the current amplitudes remaining 2, 10, and 20 s after the peak (Olivera-Bravo et al., 2007). The apparent time-to-peak was determined as the time elapsed from *I*_*ACh*_ onset to the *I*_*ACh*_ peak. We have called this parameter as “apparent” time-to-peak, just to indicate that these values do not necessarily reflect “real” time-to-peak values of nAChR activation but those observed in our experimental conditions.

To characterize the pharmacological profile of DEA, nAChRs were activated by different ACh concentrations either alone or co-applied with DEA (at roughly its IC_50_), just directly or after pre-application of DEA for 12 s. Dose-response data were fitted to the following form of the Hill equation:

(3) $$\begin{array}{l} I∕I_{max}={\left[{1+\left({EC}_{50}∕\left[ACh\right]\right)}^{n_{H}}\right]}^{-1} \end{array}$$

where *I* is the *I*_*ACh*_ peak elicited at a given *ACh* concentration (applied either alone or together with DEA); *I*_*max*_ is the maximum *I*_*ACh*_ recorded; *EC*_**50**_ is the agonist concentration required to obtain one-half the maximum *I*_*ACh*_; and *n*_*H*_ is the Hill coefficient.

Net *i/v* curves for *I*_*ACh*_ were obtained subtracting, for each voltage, the steady-state currents attained in ARN (measured during the last 100 ms of the pulse) from the corresponding ones recorded in presence of ACh. These net *I*_*ACh*_ values were normalized, for each oocyte, to the ACh response at −60 mV. The percentage of *I*_*ACh*_ inhibition at different membrane potentials (*V*_*m*_) was computed using the following equation:

(4) $$\begin{array}{l} {In}_{V_{m}}=\left[1-\left(I_{{\left(ACh+DEA\right)\;at\;V}_{m}}∕I_{{\left(ACh\right)\;at\;V}_{m}}\right)\right]\times 100 \end{array}$$

where *In*_*Vm*_ is the percentage of *I*_*ACh*_ inhibition at the corresponding *V*_*m*_; *I*_(*ACh*+*DEA*)_ _*at*_ _*Vm*_ is the *I*_*ACh*_ amplitude in the presence of ACh and DEA at *V*_*m*_; and *I*_(*ACh*)_ _*at*_ _*Vm*_ is the *I*_*ACh*_ elicited by ACh alone at *V*_*m*_. Values from the *i/v* relationship obtained at different blocker concentrations were fitted to Equations (5) and (6) to estimate the fraction of voltage field (δ) sensed by DEA at its binding site inside the channel. The apparent *K*_*i*_ for each membrane potential, which is the concentration of DEA that reduces *I*_*ACh*_ amplitude to the half, was estimated from the following equation:

(5) $$I_{ACh\;+\;DEA}/I_{ACh}=I_{min}\;+\;[\left(I_{max}-I_{\text{min}}\right)/(1+{\left([DEA]/K_{i}\right)}^{n})]$$

where *I*_*ACh*+*DEA*_ is the current evoked by co-application of 10 μM ACh with a given concentration of DEA; *I*_*ACh*_ is the current elicited by 10 μM ACh alone (control conditions); *I*_*min*_ and *I*_*max*_ are, respectively, the minimum and maximal fractional-current amplitudes evoked; *[DEA]* is the DEA concentration; and *n* is the slope factor. To estimate the fraction of the voltage field experienced by the blocking particle (δ), we used the following form of the Woodhull equation (Woodhull, 1973):

(6) $$\begin{array}{l} {logK}_{i}\left(V\right)={logK}_{i}\left(0mV\right)+\left(z\delta FV∕2.303RT\right) \end{array}$$

where *K*_*i*_ *(V)* is estimated from Equation (5) at each membrane potential; *K*_*i*_ *(0 mV)* is the *K*_*i*_-value at 0 mV; *z* is the electric charge of DEA and *R, T*, and *F* have the usual thermodynamic meanings.

Unless otherwise specified, values given are the mean ± SEM; “*n*” indicates the number of oocytes and “*N*” is the number of donors from which data were obtained. When comparing two-group means of normally distributed values, the Student's *t*-test was used; otherwise, Mann-Whitney rank-sum test was applied. Among-group differences for non-normally distributed data were determined by the Kruskal–Wallis analysis of variance on ranks and multiple comparisons vs. a control group were carried out with the Dunn's method. A significance level of *p* < 0.05 was considered for all cases.

### Virtual docking assays

The structure of the full domains of *Torpedo* nAChR was taken from RCSB Protein Data Bank (code 2BG9), which was determined, in the closed channel state, by electron microscopy at 4 Å resolution (Unwin, 2005). The edition of the protein was made using DeepView v4.1 (Guex and Peitsch, 1997) and Yasara (Krieger et al., 2002, 2004) software without further optimization. Lidocaine and DEA structures (CID 8021 and 3676, respectively) were taken from NCBI Pubchem database (http://www.ncbi.nlm.nih.gov/pccompound). A global docking procedure was accomplished with AutoDock 4 (Morris et al., 2008) implemented in Yasara, where a total of 500–1000 flexible docking runs were set and clustered around the putative binding sites. The program then performed a simulated annealing minimization of the complexes, which moved the structure to a nearby stable energy minimum, by using the implemented AMBER 99 force field (Duan et al., 2003). The binding energy was obtained by calculating the energy at infinite distance between the ligand and the nAChR oligomer and subtracting the energy of the whole complex. The more positive the binding energy, the more favorable was the interaction in the context of the force field. The best binding energy complex in each cluster was stored, analyzed and used to select the best orientation of the interacting partners. Figures were drawn with open source Pymol (The PyMOL Molecular Graphics System, Version 1.8 Schrödinger, LLC, at http://www.pymol.org/). Yasara pH command was set to 7, ensuring that molecules preserve their pH dependency of bond orders and protonation patterns. In this way, almost all DEA molecules remained charged during the docking procedure, but only 86% of the lidocaine molecules.

### Drugs

ACh, atropine sulfate, DEA, MS-222, penicillin, and streptomycin were from Sigma (St. Louis, MO, USA). HEPES was obtained from Acros Organics (New Jersey, NJ, USA). Reagents of general use were purchased from Scharlau Chemie SA (Barcelona, Spain). All solutions were made in ANR just before each application, unless otherwise stated.

## Results

### Inhibition of *I*_*ACh*_ by DEA

In either uninjected cells or oocytes bearing nAChRs, with the membrane potential held at −60 mV, DEA superfusion did not modify their membrane conductance, even at concentrations as high as 10 mM (not shown). However, in oocytes bearing nAChRs, co-application of 10 μM ACh with 0.1 μM–10 mM DEA reversibly inhibited *I*_*ACh*_s in a concentration-dependent manner (Figure 1B). The percentage of *I*_*ACh*_, normalized to the control value obtained in presence of 10 μM ACh alone, was plotted vs. the logarithm of DEA concentration, and values were fitted to a sigmoid curve (Figure 1C). The half inhibitory DEA concentration (*IC*_**50**_) was 68 μM (range 60–102 μM), similar to that found for lidocaine (73 μM, range 62–83 μM; Alberola-Die et al., 2011), and the Hill coefficient (*n*_*H*_) was 1.15 ± 0.04, suggesting that a single DEA molecule caused lidocaine-like nAChR blockade.

*I*_*ACh*_ recovery after DEA (100 μM) superfusion was slow and *I*_*ACh*_ did not reach control values even 7 min after DEA washout. So, the percentage of *I*_*ACh*_ recovery, estimated from Equation (1), was 73 ± 8% (*n* = 7, *N* = 6; Figure 1D) and 86 ± 2% (*n* = 16, *N* = 11; not shown) at 20 s and 7 min, respectively, indicating that a fraction of DEA molecules remains bound to the nAChR several minutes after DEA rinsing out.

### DEA has no effect on *I*_*ACh*_ desensitization and the apparent time-to-peak

**Figure 2:**
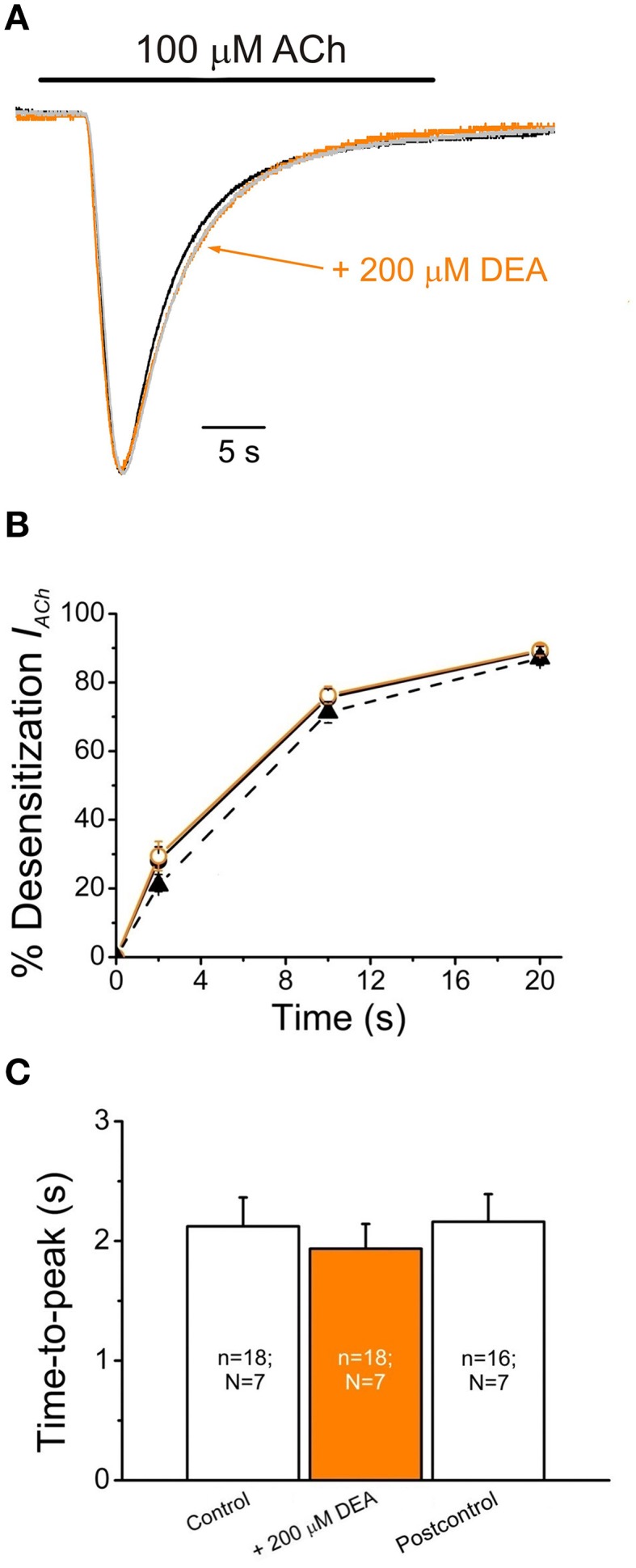
**DEA neither affects ***I***_***ACh***_ desensitization nor apparent time-to-peak. (A)** Superimposed *I*_*ACh*_s elicited by application of 100 μM ACh either alone (Control, black recoding) or together with DEA (+200 μM DEA, orange recording), and by re-applying 100 μM ACh alone 7 min after DEA washout (Postcontrol, gray recording). Note that *I*_*ACh*_ amplitudes have been scaled to the same size to better showing *I*_*ACh*_ desensitization. **(B)** Plots showing the percentage of *I*_*ACh*_ desensitization at different times (2, 10, and 20 s) after the *I*_*ACh*_ peak. Data were computed from recordings as shown in **(A)**, by applying 100 μM ACh either alone (Control, filled circles and continuous black line; Postcontrol, filled triangles and dashed line) or plus 200 μM DEA (orange circles and line). **(C)** Column graph of the *I*_*ACh*_ apparent time-to-peak when applying 100 μM ACh alone (Control and Postcontrol) or with DEA (+200 μM DEA). The number of oocytes (*n*) and donors (N) given in each column is common to **(B,C)**.

The *I*_*ACh*_ decay was not affected in oocytes superfused with 100 μM ACh together with DEA at the *IC*_**50**_ (70 μM). As shown in Figures 2A,B, DEA did not change *I*_*ACh*_ desensitization even when DEA concentration was increased to 200 μM, roughly threefold its *IC*_**50**_. The *D*_*ti*_-values obtained at 2 and 20 s (see Equation 2 in Materials and Methods) were: 30 ± 2% and 89 ± 1% (*n* = 18, *N* = 7) for *I*_*ACh*_s elicited by ACh alone vs. 29 ± 4% and 89 ± 2% (the same cells) when ACh was co-applied with 200 μM DEA (Figure 2B; *p*>0.05, Mann-Whitney rank-sum test). Moreover, DEA at concentrations as high as 200 μM had no effect on the time elapsed from *I*_*ACh*_ onset to the *I*_*ACh*_ peak (the apparent time-to-peak was 2.1 ± 0.2 s for 100 μM ACh alone against 1.9 ± 0.2 s for 100 μM ACh plus 200 μM DEA; data are from the same cells in which desensitization was measured; *p*>0.05, Mann-Whitney rank-sum test; Figures 2A,C). DEA lack of effect on *I*_*ACh*_ desensitization and apparent time-to-peak is in sharp contrast with the action of the entire lidocaine molecule on nAChRs (Alberola-Die et al., 2011, 2013).

### Voltage-dependence of nAChR blockade by DEA

**Figure 3:**
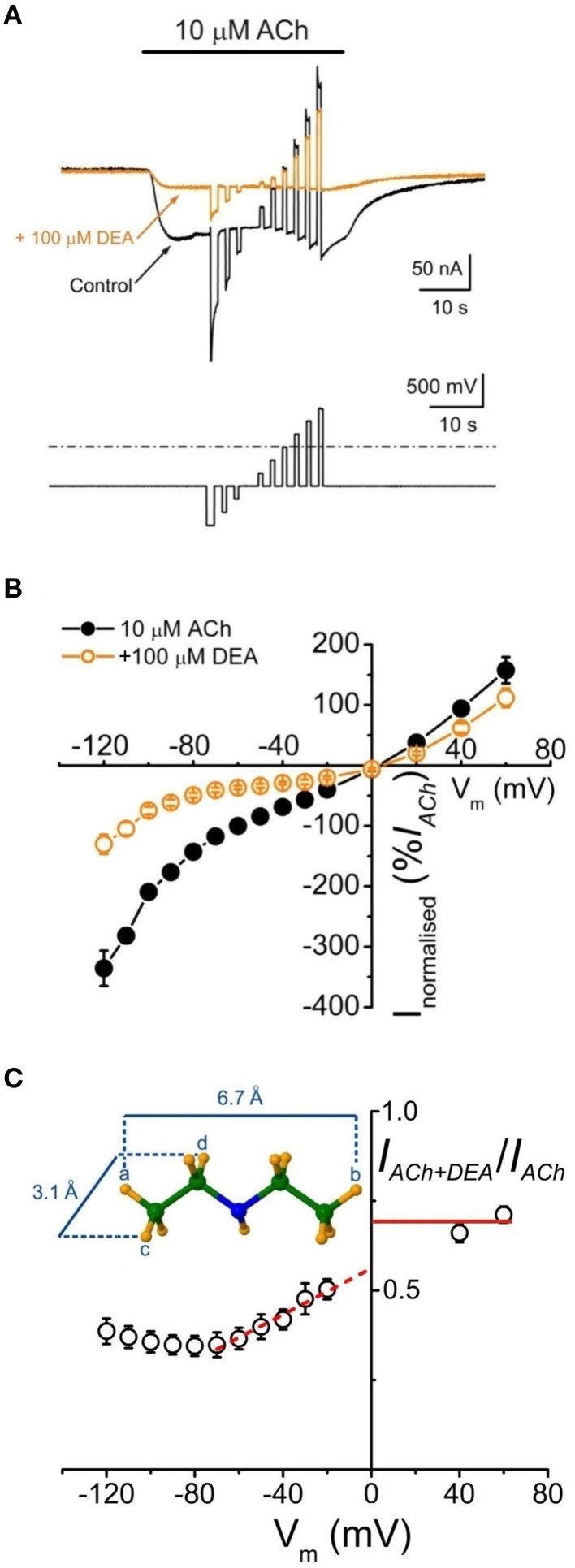
**Voltage-dependent blockade of ***I***_***ACh***_ by DEA. (A)** *I*_*Ach*_*s* (upper traces) recorded in an oocyte when applying the voltage protocol shown on bottom, during the current plateau elicited by 10 μM ACh either alone (Control, black) or together with DEA (+100 μM DEA, orange). **(B)** Net i/v relationships for *I*_*ACh*_ evoked by ACh alone (10 μM ACh, black filled circles and line) or co-applied with 100 μM DEA (+100 μM DEA, orange open circles and line), obtained when applying the voltage protocol shown in **(A)**. Normalized values represent the percentage of each *I*_*ACh*_ referred to its control at −60 mV and each point is the average of eight cells (*N* = 5). **(C)** Plot showing the fraction of the *I*_*ACh*_ left by 100 μM DEA (*I*_*ACh*+*DEA*_), normalized to its control (*I*_*ACh*_), vs. the membrane potential. Note the linear voltage dependence of *I*_*ACh*_ blockade by DEA in the range between −70 and −20 mV; the discontinuous red line shows the best linear fit to these points and the continuous line indicates the fraction of *I*_*ACh*_ remaining at positive potentials in presence of DEA. Inset shows the maximum longitudinal and transversal dimensions of the DEA molecule.

To assess whether *I*_*ACh*_ inhibition by DEA is voltage-dependent, voltage pulses were applied to oocytes while superfusing the cell with just ANR or during the *I*_*ACh*_ plateau elicited by 10 μM ACh either alone or co-applied with 100 μM DEA (Figure 3A; see Experimental Design in Materials and Methods). Figure 3B shows the *i/v* relationship obtained when plotting the net *I*_*ACh*_s elicited by ACh, either alone or with DEA, normalized to its control *I*_*ACh*_ at −60 mV, against the membrane potentials tested. The *i/v* curve for 10 μM ACh alone showed a reversal potential close to 0 mV and the characteristic inward rectification of *I*_*ACh*_ (Figure 3B, Morales et al., 1995). When DEA was co-applied with ACh the *I*_*ACh*_ reversal potential was unmodified, indicating that the channel selectivity was unaffected. However, in the presence of DEA, *I*_*ACh*_ amplitude decreased in a voltage-dependent way, the blockade being higher at negative potentials (Figure 3B). Nevertheless, the voltage dependency was only linear in the range from −70 to −20 mV, since at potentials more negative than −70 mV the *I*_*ACh*_ blockade did not increase, but even decreased. So, as displayed in Figure 3C, the fraction of *I*_*ACh*_ remaining in the presence of DEA rose as the membrane was hyperpolarized beyond −70 mV. This biphasic behavior of the voltage-dependent blockade of *I*_*ACh*_ by DEA at negative potentials was not observed for lidocaine (Alberola-Die et al., 2011), and could be due to the small size of DEA (see inset Figure 3C). Interestingly, at positive potentials DEA blocked roughly 30% of *I*_*ACh*_ (Figure 3C), indicating that DEA also causes a significant voltage-independent blockade of nAChRs.

Since DEA caused a voltage-dependent blockade of nAChRs, more evident between −70 and −20 mV (Figures 3B,C), we used in one cell the Woodhull's equation (see Equations 4–6 in Materials and Methods), restricted to this range of potentials, to estimate the δ-value, which indicates the fraction of the voltage field sensed by DEA at its binding site. The calculated δ-value was 0.30 (assuming that *z* = 1), indicating that DEA binds to the external third of the channel length.

### Effects of DEA on nAChR pharmacological profile

**Figure 4:**
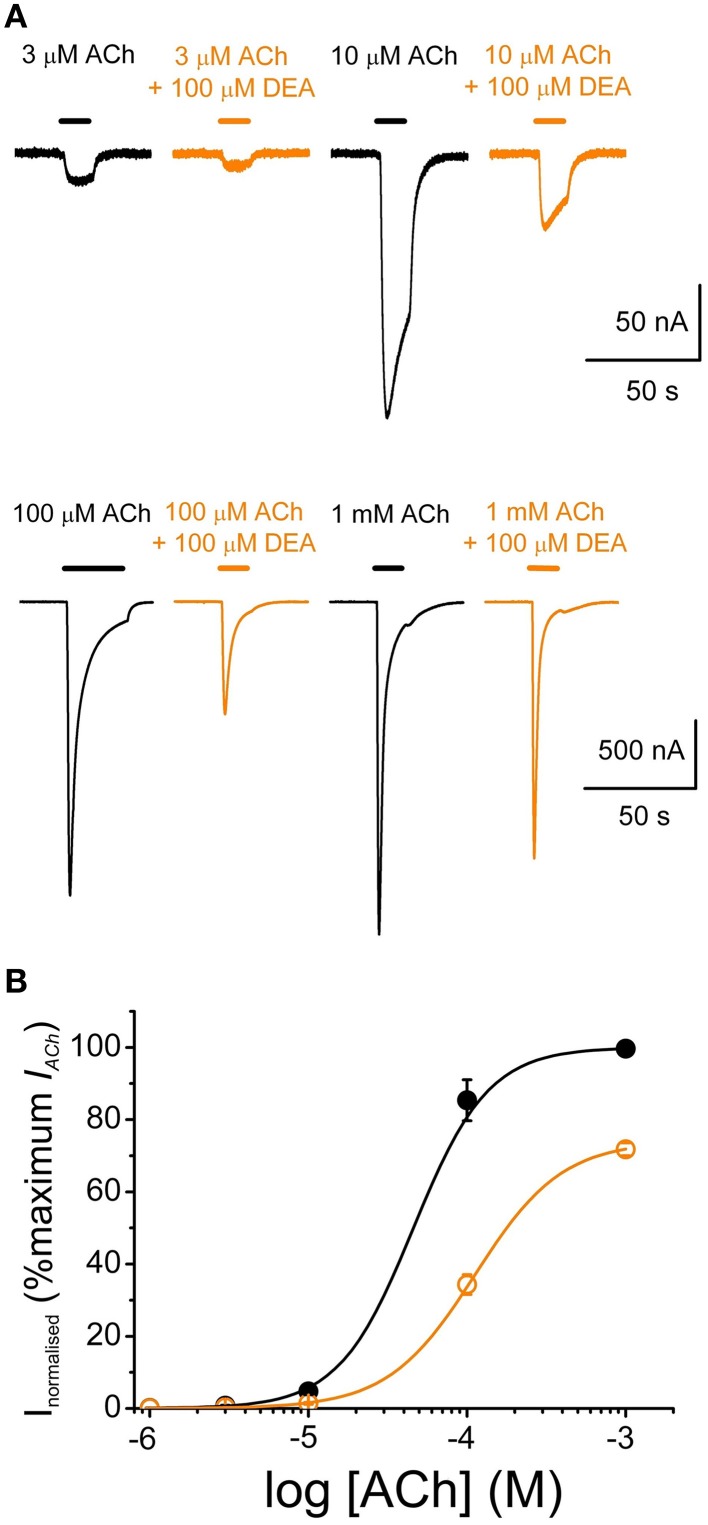
**DEA effects on ACh concentration-***I***_***ACh***_ amplitude relationship. (A)** Recordings obtained by applying sequentially to the same oocyte, at intervals of 5–30 min, increasing ACh concentrations (3–1000 μM) either alone (black) or co-applied with 100 μM DEA (orange). **(B)** Averaged ACh concentration-*I*_*ACh*_ amplitude curves for *I*_*ACh*_*s* elicited by ACh either alone (black filled circles; *n* = 4, *N* = 3) or plus 100 μM DEA (orange open circles; same oocytes). Data were normalized to the maximal *I*_*ACh*_ elicited by ACh alone and fitted to the Hill equation (continuous lines).

**Figure 5:**
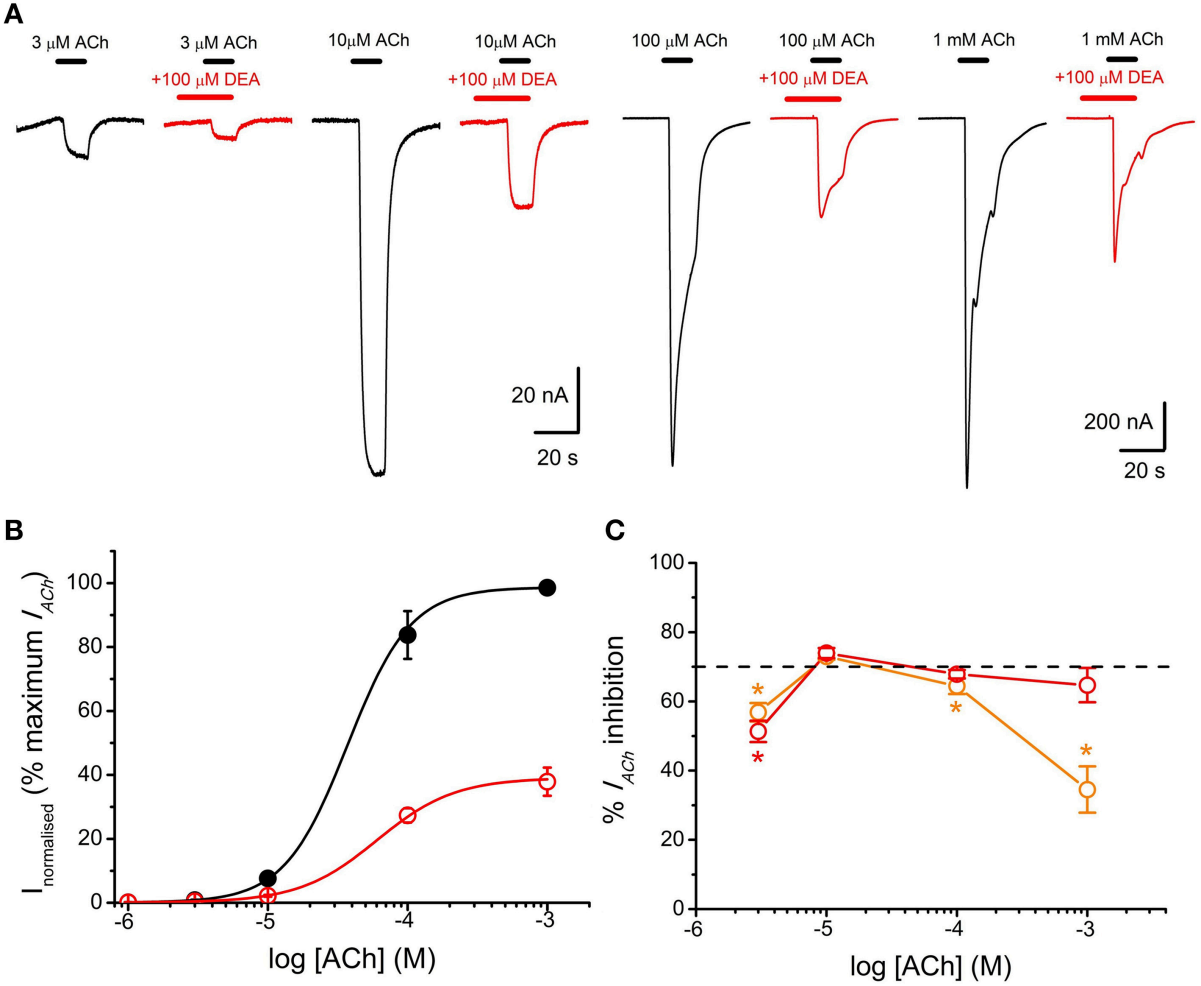
**DEA pre-application increases ***I***_***ACh***_ inhibition and changes the pharmacological profile of nAChR inhibition. (A)** *I*_*ACh*_s elicited by pre-application of 100 μM DEA for 12 s followed by its co-application with ACh at the indicated concentrations (red recordings) or by just ACh at the same concentrations (black recordings). **(B)** Averaged ACh concentration-*I*_*ACh*_ amplitude curves for *I*_*ACh*_*s* evoked by ACh either alone (black filled circles; *n* = 4–8, *N* = 1–3) or co-applied with 100 μM DEA, after 12 s pre-application of DEA at the same concentration (red open circles; same oocytes). Data were normalized to the maximal *I*_*ACh*_ elicited by ACh alone and fitted to the Hill equation (continuous lines). **(C)** Plot showing the percentage of *I*_*ACh*_ inhibition when ACh (at different concentrations) was directly co-applied with 100 μM DEA (orange circles; *n* = 5–29, *N* = 4–10), or when ACh and DEA co-applications were preceded by 100 μM DEA pre-application for 12 s (red circles; *n* = 7–11, *N* = 1–3). Asterisks indicate significant differences (*p* < 0.05, Kruskal Wallis ANOVA on ranks) respect to the *I*_*ACh*_ blockade caused by solely co-applying 10 μM ACh and 100 μM DEA; the dashed line indicates 70% inhibition. Note that the percentage of *I*_*ACh*_ inhibition decreased markedly when DEA was just co-applied with high ACh concentrations. By contrast, when DEA was pre-applied before its co-application with ACh, the percentage of *I*_*ACh*_ blockade was similar at the different ACh concentrations (see text). The slight decrease in *I*_*ACh*_ blockade by DEA observed at 3 μM ACh might not be reliable because of the inaccuracies own to the small size of *I*_*ACh*_ at this agonist concentration.

To study the effects of DEA on the ACh dose-*I*_*ACh*_ relationship, ACh was applied in the same oocyte at increasing concentrations (1 μM–1 mM) either alone or with 100 μM DEA (Figure 4A). Figure 4B shows the ACh dose-*I*_*ACh*_ curves obtained either in absence or in presence of 100 μM DEA, normalizing *I*_*ACh*_ values to those evoked by 1 mM ACh alone; data were fitted to sigmoid curves with the Hill equation (see Equation 3 in Materials and Methods). The *EC*_**50**_ for the control curve (ACh alone) was 46 μM (range 16–59 μM) and the *n*_*H*_ 1.8 ± 0.1 (*n* = 4, *N* = 3), values which are in good agreement with previous data (Morales et al., 1995; Olivera-Bravo et al., 2005). In presence of 100 μM DEA, the maximum *I*_*ACh*_ amplitude decreased, suggesting a non-competitive blockade (Figures 4A,B); besides, the dose-response curve shifted to the right, increasing the *EC*_**50**_ more than twice the original value (112 μM, range 88–145 μM) and slightly decreasing the *n*_*H*_ to 1.6 ± 0.1 (Figure 4B). As shown in Figures 4A, 5C, co-application of 10 μM ACh with 100 μM DEA caused an *I*_*ACh*_ inhibition of 73 ± 1% (*n* = 29, *N* = 10), but when the same concentration of DEA was co-applied with 1 mM ACh, the percentage of blockade was only 35 ± 7% (*n* = 5, *N* = 4; *p* < 0.05, *t*-test). Thus, the percentage of *I*_*ACh*_ inhibition by DEA markedly depended on the ACh concentration, suggesting that the blocking effect of DEA on nAChRs was not merely non-competitive. This apparently competitive inhibition of *I*_*ACh*_ by DEA could be explained, at least partially, by its binding to resting nAChRs. To test this hypothesis, we determined the percentages of *I*_*ACh*_ blockade induced by DEA (100 μM) when it was pre-applied to the cell for 12 s before being co-applied with ACh at increasing concentrations (1 μM–1 mM; Figure 5A). Figure 5B shows the ACh dose-*I*_*ACh*_ curves elicited by ACh either alone or when it was co-applied with100 μM DEA after its pre-application for 12 s. In these experiments, the *EC*_**50**_ for ACh alone was 37 μM (range 14–115 μM) and the *n*_*H*_ 1.9 ± 0.1 (*n* = 7–11, *N* = 1–3) whereas in presence of DEA the *EC*_**50**_ slightly increased, to 60 μM (range 52–126 μM), and the *n*_*H*_ was 1.6 ± 0.1. Noticeably, when DEA was pre- and co-applied with ACh, a smaller maximum *I*_*ACh*_ amplitude was reached (Figures 5A,B) and the percentage of *I*_*ACh*_ inhibition caused was similar for any ACh concentration above 10 μM (*p*>0.05, Kruskal Wallis ANOVA on ranks; red plot of Figure 5C), indicating a non-competitive blockade of nAChRs. Thus, the effect of DEA pre- and co-application strikingly contrasted with that obtained by just DEA co-application with ACh, since the latter showed an apparent competitive mode of action while the former did not (Figures 4B, 5C). If DEA binds to, and blocks, closed (resting) nAChRs, it would be expected that DEA pre-application enhanced the *I*_*ACh*_ inhibition caused by just DEA and ACh co-application, preferentially at the highest ACh doses (1 mM), as we have indeed observed (Figure 5C). This is likely due to the fast activation of most nAChRs by 1 mM ACh, which prevented the effect of DEA on resting nAChRs when ACh and DEA were just co-applied. On the other hand, at low ACh doses only a small percentage of nAChRs were activated by the agonist and consequently most nAChRs remained resting and therefore susceptible to be blocked by DEA, acting as a closed-channel blocker.

### Virtual docking assays of DEA- and lidocaine-nAChR interactions

Virtual docking assays were carried out to explore DEA- and lidocaine-nAChR interactions using as template the full structure of *Torpedo* nAChRs (see Materials and Methods). For these assays 1000 runs of interactions were performed for DEA and 500 for lidocaine molecules.

**Figure 6:**
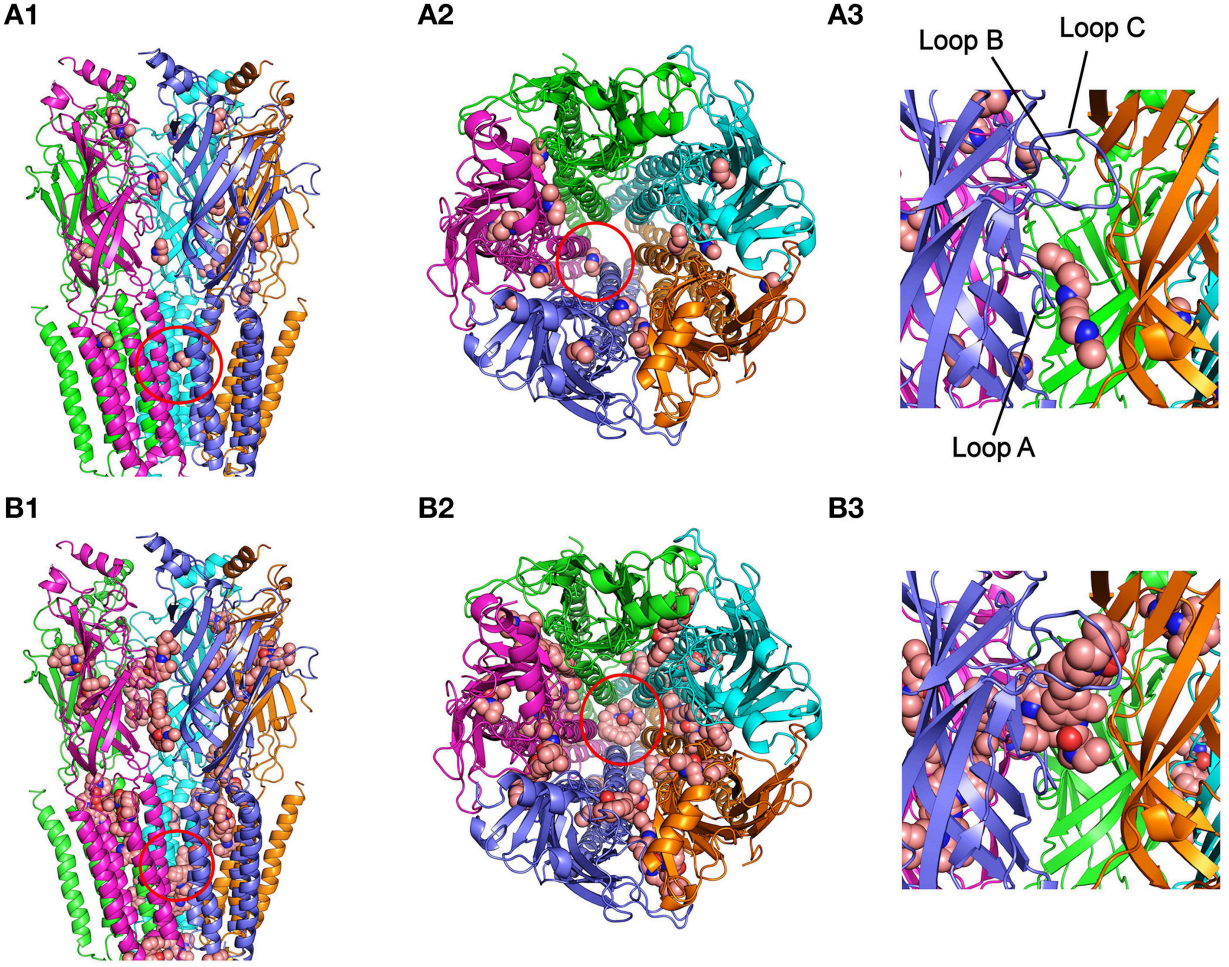
**Modeling of DEA and lidocaine binding to the EC and TM domains of the nAChR. (A_**1**_)** Lateral view, in the membrane plane (top corresponding to the synaptic cleft), of nAChR with bound DEA molecules. For this and following panels, the color code for nAChR subunit is: α (blue and cyan), β (magenta), γ (orange), and δ (green). Ligand molecules are colored brown and represented in van der Waals spheres. Note that very few DEA-nAChR binding solutions (clusters) were located at the TM domain, except two inside the channel pore (red circle), but there were several clusters distributed at the EC domain. **(A**_2_**)** Upper view, from the synaptic cleft, of the nAChR with bound DEA. Note that at the EC domain DEA mainly interacts at intersubunit interphases, although some DEA clusters involved single subunits. The red circle indicates the cluster sited within the channel pore. **(A**_3_**)** An expanded view of the EC domain at the α-γ interphase, corresponding to the ACh-binding site (loops A, B, and C of the α subunit are indicated as reference). Note that one DEA cluster is near, but not inside, the pocket of the ACh-binding site (see text for details). **(B**_1_**)** nAChR with lidocaine binding solutions is shown in a similar view as in **(A**_1_**)**. Note that lidocaine clusters were more numerous at the TM domain than those observed for DEA. Several TM clusters were located in intra- and intersubunit cavities and others inside the channel pore (red circle). At the EC domain there were also several lidocaine clusters, with a distribution roughly similar to that found for DEA. **(B**_2_**)** Upper view, from the synaptic cleft, of the nAChR with bound lidocaine. Note that both at the EC and TM domains lidocaine interacted at intra and intersubunit interphases and that several clusters were grouped inside the channel pore (red circle). **(B**_3_**)** The same nAChR area as in **(A**_3_**)**, showing a lidocaine cluster into the ACh-binding site.

The runs for DEA-nAChR interactions disclosed 30 clusters of sites that differed in less than 5 Å of root-mean-square-deviation of which 23 (77%) corresponded to EC, 2 (7%) to TM, and the remaining 6 (16%) to intracellular domains; this latter solutions have been discarded for further analysis because DEA is impermeable through the cell membrane and so they lack of functional meaning. As shown in Figure 6 (upper panels), the small and polar DEA barely binds to the TM domain, except inside the channel pore (Figures 6A_1_,A_2_), but it interacted quite well with different nAChR residues located at the EC domain (Figures 6A_1_,A_3_). Regarding the EC domain, most DEA-clusters were detected at α_γ_, β, or γ subunits (Figures 6A_1_,A_2_). Interestingly, some DEA clusters were located at the α-γ interphase, in a cavity near the orthosteric binding site (Figure 6A_3_). In fact, one of those clusters involved the Y93 (loop A) of the α subunit and the D176 (loop F) of the γ subunit, although due to the small size of DEA this cluster did not reach neither the B nor the C-loop (Figure 6A_3_), which also contribute with key residues to the ACh binding pocket (Corringer et al., 2000). By contrast, the docking-assays did not show any equivalent hotspot for DEA binding at the interphase of α-δ subunits (see Figure 6A_2_). The remaining DEA clusters at the EC domain were mostly at intersubunit crevices, although a few solutions involved a single chain of α, β, or γ subunits (Figures 6A_1_,A_2_). At the TM domain, we only detected 2 DEA clusters located near and into the channel pore. Both clusters involved interactions with α-β or α-δ subunits (Figure 6A_2_ and Supplementary Figure 2) and their binding sites were at a depth circa one third of the whole membrane thickness, from the extracellular side (see Supplementary Figure 2).

For lidocaine-nAChR interactions, the docking assays revealed multiple hotspots both at EC and TM domains (Figure 6, lower panels), being remarkable the large number of solutions found at the TM domain as compared with DEA. At the EC region, it is noteworthy that one of the lidocaine clusters fitted quite well at the nAChR ligand binding sites in the α-γ (Figures 6B_2_,B_3_) and the α-δ interphases (Figure 6B_2_), involving interactions with key residues of A, B, and C loops of the α subunit. In contrast to DEA, the adjustment of this cluster to the ACh-binding site is possible because the longitudinal and transversal molecular dimensions of lidocaine (10.7 and 4.8–6.7 Å, respectively) are relatively close to those of the ACh molecule (9.1 and 3.7 Å, respectively). Besides, some lidocaine clusters at the EC domain were located at intersubunit crevices and, less frequently, at intrasubunit sites, with a roughly similar pattern to that found for DEA (compare Figure 6A_1_ and Figure 6B_1_). At the TM domain, lidocaine clusters could be grouped in three main types of interactions: (i) inside the pore of the channel, where lidocaine contacted residues of M2 segments from four or five subunits; (ii) intrasubunit interactions, with low binding energy, at the cavities among the M1, M2, M3, and M4 segments of each subunit; and (iii) intersubunit interactions, where α-γ, α-δ, and β-δ hotspots presented the strongest interactions with lidocaine.

## Discussion

Lidocaine is an aminoethylamide local anesthetic that in physiological solutions exists as a mixture of charged and uncharged species, due to its pK_a_ of 7.8 (Liu et al., 2003). Though it is believed that the charged form of lidocaine mediates most of its therapeutic action (Narahashi et al., 1969), the neutral form is also important because of its higher ability to penetrate inside membranes. Interestingly, both charged and uncharged forms of lidocaine seem to contribute to the complex inhibitory action of this molecule on muscle- (Alberola-Die et al., 2011) and neuronal-type (Alberola-Die et al., 2013) nAChRs. Now, we report the effects of DEA, a small amine mimicking the hydrophilic moiety of lidocaine, on muscle-type nAChR, which is mostly protonated at physiological pH, since it has a pK_a_ of 10.49 (Sergeeva et al., 2000).

DEA effects on nAChRs reported here showed important analogies with those described for lidocaine. So, the *IC*_**50**_ for DEA (68 μM) was quite similar to that found for lidocaine (73 μM; Alberola-Die et al., 2011); besides, DEA and lidocaine blocked nAChRs in a voltage-dependent manner, but also bound to resting (closed) nAChRs. However, there were important differences between lidocaine and DEA actions on nAChRs, mainly in the desensitization rate, which was markedly increased by lidocaine (Alberola-Die et al., 2011), but unaffected by DEA, even at concentrations well over its *IC*_**50**_. This differential effect is of great functional relevance because it points out that the faster *I*_*ACh*_ decay induced by lidocaine is indeed caused by an enhancement of nAChR desensitization, rather than by a slow nAChR blockade.

It has been previously shown that DEA, and also triethylamine, can directly activate muscle-type nAChRs, and so act as partial agonists (Sánchez et al., 1986). However, this effect was observed at concentrations far higher (mM range) than those used here to characterize DEA inhibitory actions on nAChRs. In our hands, such high doses would almost completely block the *I*_*ACh*_ (Figure 1). Besides, in oocytes bearing nAChRs, we could not detect any current activated by DEA alone, even when applied up to 10 mM. Thus, although it cannot be fully ruled out that DEA causes some competitive inhibition on nAChRs, the marked shift to the right of the ACh dose-*I*_*ACh*_ curve (Figure 4) rather suggests the involvement of other mechanisms (i.e., blockade of closed nAChRs, as indicated below). We have also rule out that DEA caused an unspecific blockade of LGICs, since 100 μM DEA showed no effect on GABA-elicited currents from rat neuronal GABA_*A*_Rs microtransplanted to *Xenopus* oocytes (see Supplementary Figure 1), which belong to the same family of LGICs. Nevertheless, it remains to be determined whether or not DEA has specific effects or different potencies on the neuronal nAChR subtypes.

DEA, as the entire lidocaine molecule, inhibited nAChRs in a voltage-dependent manner, the blockade being higher at negative potentials, which strongly suggests an open-channel blockade caused by this positively-charged molecule. However, at potentials more negative than −70 mV, the *I*_*ACh*_ remaining upon DEA did not decrease but rather increased (Figure 3C), in contrast to the voltage-dependent effect of lidocaine, which kept increasing at very negative potentials (Alberola-Die et al., 2011). This difference might arise because of the smaller size of DEA, which could allow its permeation through the channel pore, by a “punch-through” mechanism, as it has been proposed for other small molecules in this receptor (Sine and Steinbach, 1984). In fact, from data on permeation of various sized cations, the estimated size of the pore was 8.4 Å (Cohen et al., 1992), which agrees well with the 9–10 Å pore diameter estimated from images of the open state of *Torpedo* nAChRs (Unwin, 1995). Given the small size of DEA (smaller than 7 Å in the long axis; see Figure 3C) and its positive charge in physiological solutions, this molecule could be electrostatically forced to move from its binding site to the cytoplasm, releasing the nAChR from its blockade at highly negative potentials. Virtual docking assays also showed that both DEA and lidocaine might bind inside the channel pore (Figure 6), though lidocaine protruding more into it (Figure 6 and Supplementary Figure 2). The predicted DEA binding sites within the pore were slightly shallower than the lidocaine loci, in spite that for both molecules the estimated δ-values were similar (about 30% of the electrical field). Nevertheless, interpretation of the electrical distances (δ) is not always straightforward because the IC_50_s are dependent not only on the equilibrium dissociation constants of the blockers, but also on the kinetics of the transitions between functional states (Pascual and Karlin, 1998). Interestingly, whereas lidocaine could interact within the pore at all five subunits, DEA interacted with residues belonging to just two subunits (α-β or α-δ), which might cause fainter interactions and thus facilitate the punch-through occurrence.

*I*_*ACh*_ blockade by DEA could not be fully reversed by applying pulses to positive potentials, which should unplug the positively charged DEA from the channel pore. Thus, at +60 mV, the *I*_*ACh*_ inhibition caused by 100 μM DEA (roughly 30%) was voltage-independent (Figure 3C) and, so, it is not mediated by open-channel blockade of nAChRs. Consequently, DEA should also bind outside the pore and cause the blockade of resting nAChRs, as it has been proposed for other non-competitive blockers of nAChRs, including some cholinesterase inhibitors, such as tacrine (Prince et al., 2002) or edrophonium (Olivera-Bravo et al., 2007) and local anesthetics, such as tetracaine (Papke and Oswald, 1989; Gallagher and Cohen, 1999; Middleton et al., 1999) or lidocaine (Alberola-Die et al., 2011). DEA binding to closed nAChRs is also supported by its pronounced inhibitory action on nAChRs when it is pre-applied to the cells before being co-applied with ACh (Figure 5). This was particularly evident at high ACh doses (Figure 5C), since the fast opening of nAChR channels by ACh prevented the DEA block of resting receptors when DEA and ACh were solely co-applied. As DEA caused nAChR blockade at positive potentials and it is permanently charged (preventing their permeation through the membrane), it is suggested that DEA blocked resting nAChRs by its binding to extracellular residues outside the pore, which differs from the binding of tetracaine within the channel on resting nAChRs (Gallagher and Cohen, 1999; Middleton et al., 1999). Accordingly, our virtual docking results showed that both DEA and lidocaine bind at multiple loci on the EC domain, including several sites at intersubunit interphases. Of particular functional relevance seems the DEA binding to a cavity near the ligand-binding pocket at the α-γ interphase (Figure 6A_3_). Interestingly, this DEA binding site is very similar to that found for ketamine binding to the homologous GLIC channel, as resolved by X-ray crystallography at 2.99 Å (Pan et al., 2012; compare panels A_1_ and A_2_ of Supplementary Figure 2). It is also remarkable that ketamine, a small water soluble amine, blocked GLIC channels with an IC_50_ of 58 μM, which is very close to that of DEA for nAChRs blockade (Figure 1). Even more, ketamine also blocked, at this range of concentrations, mammalian muscle- and neuronal-type nAChRs by acting on the open and closed states (Scheller et al., 1996), although it neither blocked GABA_A_R nor GlyRs (Yamakura et al., 2000). Therefore, since DEA, and most likely ketamine, binding site at the α-γ interphase of nAChRs overlaps partially with the ACh-binding pocket, it might additionally inhibit nAChR by competitive antagonism. Finally, our docking results do not support a role of DEA as a partial nAChR agonist because of, at least partially, its small size. Thus, although DEA interacted with A-loop residues from the α-subunit, this binding site was 15–18 Å below the C loop, which is known to contribute with key residues to the ligand-binding site (Corringer et al., 2000). Moreover, the docking-assays did not show any equivalent hotspot for DEA binding at the interphase of α-δ subunits. By contrast, lidocaine interacted with residues from all α loops conforming the ACh-binding pocket, both at the α-γ and α-δ interphases, which suggests a possible additional action as a competitive antagonist.

In conclusion, we found that DEA, a small charged molecule closely resembling the hydrophilic moiety of the lidocaine molecule, mimics some, but not all the inhibitory actions of the complete lidocaine molecule on nAChRs. So, DEA accounts for the voltage-dependent blockade of nAChRs by lidocaine and might also contribute to inhibit resting nAChRs, likely acting on extracellular residues lying outside the channel pore.

## Author contributions

All authors listed, have made substantial, direct and intellectual contribution to the work, and approved it for publication.

## Funding

This work was supported by grants BFU2012-31359, BFU2012-39092-C02-01, BFU2011-25920, and CSD2008-00005 from the MINECO and PROMETEO/2014/11 from GVA (Spain).

### Conflict of interest statement

The authors declare that the research was conducted in the absence of any commercial or financial relationships that could be construed as a potential conflict of interest.

## Abbreviations

ACh: acetylcholine
ANR: normal Ringer solution with atropine
DEA: diethylamine
EC domain: extracellular domain
ELIC: *Erwinia chrysanthemi* ligand-gated ion channel
GABA_A_R: gamma-aminobutyric acid type A receptors
GLIC: *Gloebacter violaceus* ligand-gated ion channel
GlyR: glycine receptor
*I*_*ACh*_: ACh-elicited current
LA: local anesthetic
LGIC: ligand gated ion channel
MS-222: ethyl 3-aminobenzoate methanesulfonate
*n*: number of oocytes
*N*: number of oocyte donors
nAChR: nicotinic acetylcholine receptor
NR: normal Ringer solution
TM: transmembrane segment.
