# Supplementary material for: Muscle-Type Nicotinic Receptor Blockade by Diethylamine, the Hydrophilic Moiety of Lidocaine
Source: Front Mol Neurosci. 2016 Feb 15;9:12. doi: 10.3389/fnmol.2016.00012 (PMC4753328; doi:10.3389/fnmol.2016.00012)
Supplement: Supplementary file 1 [file Image1.PDF]

Supplementary Material

Muscle-type nicotinic receptor blockade by diethylamine, the hydrophilic moiety of lidocaine

Armando Alberola-Die[1], Gregorio Fernández-Ballester[2], José Manuel González-Ros[2], Isabel Ivorra[1], Andrés Morales[1]*

[1]División de Fisiología, Departamento de Fisiología, Genética y Microbiología, Universidad de Alicante, Apdo. 99, E-03080 Alicante, Spain.
[2]Instituto de Biología Molecular y Celular, Universidad Miguel Hernández, Elche, E-03202, Alicante, Spain*

Correspondence: Andrés Morales: andres.morales@ua.es

FIGURE 1:

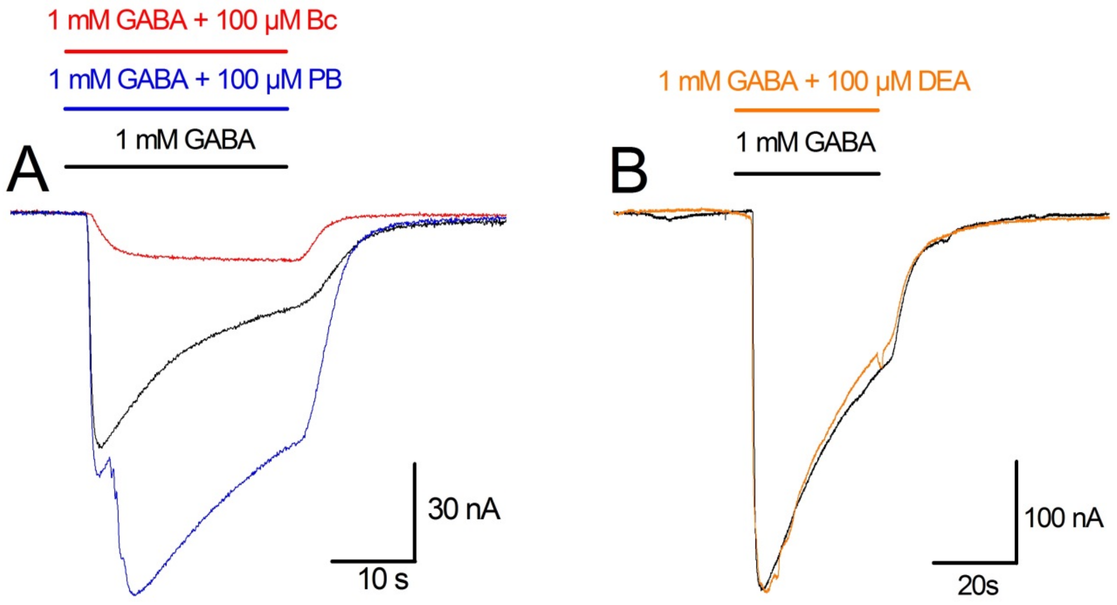

Supplementary Figure 1. *Lack of effect of DEA on GABA-elicited currents.* $GABA_A$ receptors ($GABA_A$Rs) were microtransplanted to the *Xenopus* oocyte membrane from rat-brain synaptosomal-enriched membranes (see below the protocol used to obtain these membranes). (A) GABA-elicited currents were recorded, at a holding potential of -60 mV, by superfusing the cell with 1 mM GABA

either alone (control, black recording) or together with 100 µM pentobarbital (PB; blue recording) or 100 µM bicuculline (Bc; red recording), to potentiate or inhibit, respectively, $GABA_A$Rs. Notice that microtransplanted $GABA_A$Rs retained their characteristic functional properties in the *Xenopus* oocyte membrane. (B) Interestingly, 100 µM DEA neither affected the current amplitude nor the desensitization time-course of GABA-elicited currents, indicating that DEA effects on muscle-type nAChRs are not common to all members of the same family of LGICs. Bars above the recordings indicate the timing of the indicated drug applications.

Protocol followed to obtain rat brain synaptosomal-enriched membranes: Adult female Wistar rats (200–250 g) were anaesthetized with PB and decapitated. Animal handling was carried out in accordance with the guidelines for the care and use of experimental animals adopted by the E.U. and the animal protocol was approved by the ethic committee of Universidad de Alicante. Whole brains were removed and homogenized in a glass–Teflon homogenizer in 10 volumes of ice-cold 10 mM Tris–citrate buffer (pH 7.4) containing 300 mM sucrose, 1 mM EDTA, 1 mM EGTA and 500 µM PMSF. Each homogenate was spun at 1000 ×*g* for 10 min and the supernatant centrifuged at 45,000 × *g* for 30 min (4 °C). Pellet was resuspended in 5 ml of 50 mM Tris–citrate (pH 7.4) containing 1 mM EDTA and 1 mM EGTA and 500 µM PMSF, frozen in liquid N_2 for 5 min and then thawed for 20 min at 20 °C in an ultrasonic bath. Suspension was spun 30 min (4 °C) at 45,000 ×*g*. The final pellet was resuspended in 50 mM Tris-citrate buffer in 1:10 mass/volume ratio and protein concentration assayed (Pierce kit, Pierce Chem. Co., Rockford, IL, USA). Samples containing 1-3 mg ml^{-1} of protein were aliquoted and kept at -80 ºC for later use.

FIGURE 2:

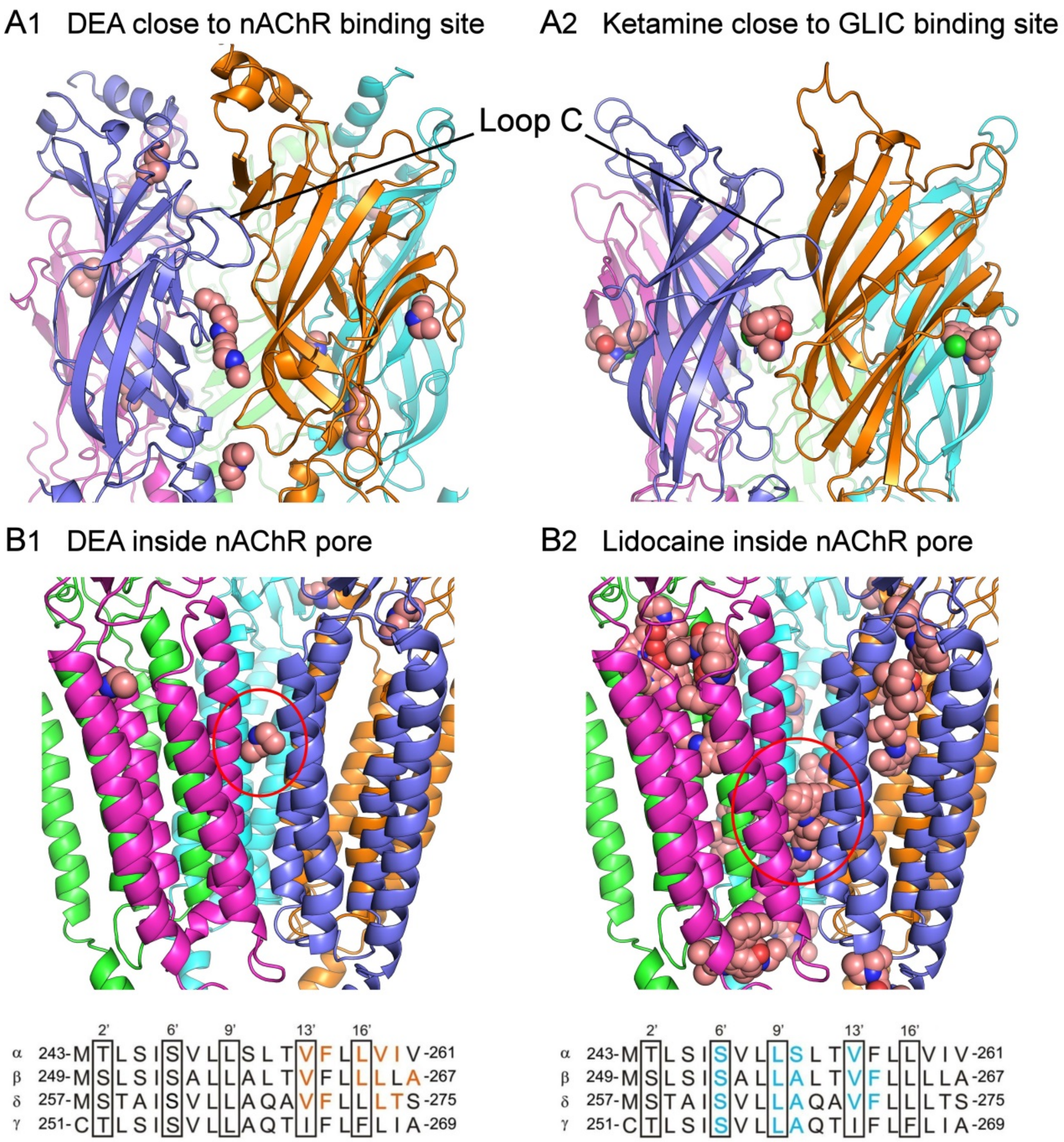

Supplementary Figure 2. *DEA binding sites at the extracellular (EC) and transmembrane (TM) domains of the nAChR as compared to those of ketamine and lidocaine*. DEA, lidocaine and ketamine molecules are coloured brown and represented in van der Waals spheres. (**A_1**) Virtual docking assays showed that *Torpedo* nAChR has a hotspot for DEA binding located at the interphase between α (blue) and γ- (orange) subunits, quite near the pocket corresponding to the orthosteric binding site. Actually, our analysis placed the binding site for DEA about 15-18 Å below the C loop, which is known to contribute with key residues to the ligand-binding site. (**A_2**) Interestingly, the X-ray structure of GLIC with bound ketamine has been resolved at 2.99 Å (code 4F8H; Pan *et al*. 2012; DOI: http://dx.doi.org/10.1016/j.str.2012.08.009) and this structure shows that ketamine binds to an

intersubunit cavity at the EC domain which is near, 9–10 Å below, the ligand-binding site. Note that this cavity has a similar location, although no identical, to that here reported for DEA at the EC of nAChRs. **(B_1-B_2)** Lateral view, in the membrane plane (top corresponding to the synaptic cleft), of M1-M4 segments of each nAChR subunit: α (blue and cyan), β (magenta), γ (orange) and δ (green). The red circles show the sites where DEA (**B_1**) and lidocaine (**B_2)** interact with M2 residues within nAChR pore, which are roughly located at 30-50% of the channel extent. The aligned amino acid sequences for the M2 segments of the 4 subunits (α, β, δ and γ) constituting the nAChR pore are represented below the nAChR. The numbers at the beginning and the end of each sequence correspond to the first and the last amino acid represented, respectively. Black frames indicate those amino acids in each subunit lining the channel pore, being closer to the intracellular side those located at 2'. Amino acids typed in orange (**B_1**) and cyan (**B_2**) correspond to those TM residues interacting with DEA and lidocaine, respectively. Note that, DEA and lidocaine share binding sites within the channel, although lidocaine besides binds to deeper residues.
